# State-wise geospatial analysis of dog bite burden in India from 2018 to 2023

**DOI:** 10.1097/j.pbj.0000000000000305

**Published:** 2025-10-20

**Authors:** Dodda Basava Janekunte, Onkar Yadav Ninganna, Poorvitha Hadya Palaksha

**Affiliations:** a Jawaharlal Nehru Medical College, KAHER, Belagavi, Karnataka, India,; b Masters in Public health, School of Public health, JSSAHER, Mysore, Karnataka, India.

**Keywords:** rabies, zoonoses, neglected tropical diseases, epidemiology

## Abstract

**Background:**

Around 59,000 rabies deaths still occur annually. According to WHO, India accounts for 36% of the global deaths due to rabies and 65% of the deaths due to rabies in the Southeast Asia region. India, being highly endemic for rabies, has the largest number of animal bites in the World. So, this study will identify the high-risk areas for dog bites in India through geospatial analysis for better outcomes.

**Objectives:**

To analyze the trends in dog bite cases in India from 2018 to 2023 and to identify the high-risk areas using geospatial analysis.

**Methods:**

A retrospective analysis was conducted using open government data on dog bite cases across Indian states and union territories from 2018 to 2023. A geospatial analysis was done year-wise to identify high-risk areas for dog bites.

**Results:**

Dog bite cases peaked in 2018 (7.57 million), followed by a steady decline until 2022 (2.18 million), with an increase in 2023 (2.76 million). Uttar Pradesh, Bihar, and Maharashtra consistently reported the highest burden.

**Conclusions:**

While India has made progress in dog bite reduction through vaccination and sterilization programs, the increase in 2023 highlights the need for sustained efforts. Strengthening surveillance, improving public awareness, and ensuring widespread access to rabies prophylaxis are crucial for long-term control. Future research should focus on socioenvironmental determinants and intervention effectiveness to refine public health strategies.

## Introduction

India, being highly endemic for rabies, has the largest number of animal bites in the World. About 15 million people are bitten by animals, mostly dogs, every year and require postexposure prophylaxis.^1,2^ Rabies is a vaccine-preventable, neglected tropical disease, and the deadliest viral zoonosis known in history.^3^ Around 59,000 rabies deaths still occur annually.^4^ According to WHO, India accounts for 36% of the global deaths due to rabies. India also accounts for 65% of the deaths due to rabies in the Southeast Asia region.^5^ The true burden of rabies in India is not fully known; although, as per available information, it causes 18,000‒20,000 deaths every year. About 30‒60% of reported rabies cases and deaths in India occur in children younger than 15 years as bites that occur in children often go unrecognized and unreported.^6^ Across Asia, the annual expenditure due to rabies is estimated to reach 563 million USD.^7^ Tripartite Alliance Food and Agriculture Organization, World Organization for Animal Health, World Health Organization (FAO, WOAH, and WHO), and the Global Alliance for Rabies Control have launched “Zero by 30: The Global Strategic Plan to End Human Deaths from Dog-Mediated Rabies by 2030.”^8^ Rabies deaths can be prevented by effective post-exposure prophylaxis (PEP) with potent rabies vaccines and immunoglobulins administered soon after the exposure.^9^ Addressing this issue requires a robust One Health approach, which emphasizes the interconnectedness of human, animal, and environmental health. Despite progress, the journey toward the goal of eliminating DMHR by 2030 is fraught with challenges, including free-roaming dog management, vaccination strategies, and intersectoral collaboration.^10^ So this study is planned to know the high-risk areas for dog bites in India through geospatial analysis for better outcomes.

## Methodology

We have used Open Government Data from 2018 to 2023 to assess the burden of dog bites in India. OGD Platform was launched in 2012, an initiative by the Government of India aimed at fostering transparency, accountability, and citizen engagement by making government data freely accessible to the public in reusable formats. Managed by the National Informatics Centre under the Ministry of Electronics and Information Technology, the initiative is primarily hosted on the Open Government Data Platform India. This platform provides datasets, documents, services, and applications contributed by various government ministries and departments, covering diverse domains such as health, education, agriculture, and transportation. The data is available in machine-readable formats like CSV, JSON, and XML, ensuring ease of use for researchers, developers, and innovators. The primary objectives of OGD India are to promote transparency, drive innovation, and enable data-driven decision-making in both public and private sectors. Users can explore datasets, use APIs for app development, and apply visualization tools to analyze and present data effectively. This initiative supports evidence-based governance, encourages research, and facilitates solutions in health care, agriculture, and urban planning, contributing significantly to India's digital governance ecosystem and the global Open Data movement.^11^ The spatial and temporal analysis of dog bite cases has been mapped statewide using QGIS LTR version 3.34.14 (Quantum Geographic Information System). The spatial analysis maps the geographic distribution of dog bite cases across regions to identify high-risk areas, while the temporal analysis detects trends over time, including seasonal patterns or changes in incidence.

The study areas we have taken to analyze the distribution of dog bites in India from 2018 to 2023 include the following states and union territories: Andaman and Nicobar Islands, Andhra Pradesh, Arunachal Pradesh, Assam, Bihar, Chandigarh, Chhattisgarh, Dadra and Nagar Haveli, Daman and Diu, Delhi, Goa, Gujarat, Haryana, Himachal Pradesh, Jammu and Kashmir, Jharkhand, Karnataka, Kerala, Ladakh, Lakshadweep, Madhya Pradesh, Maharashtra, Manipur, Meghalaya, Mizoram, Nagaland, Odisha, Puducherry, Punjab, Rajasthan, Sikkim, Tamil Nadu, Telangana, Tripura, Uttar Pradesh, Uttarakhand, and West Bengal.

## Results

**Figure 1.**
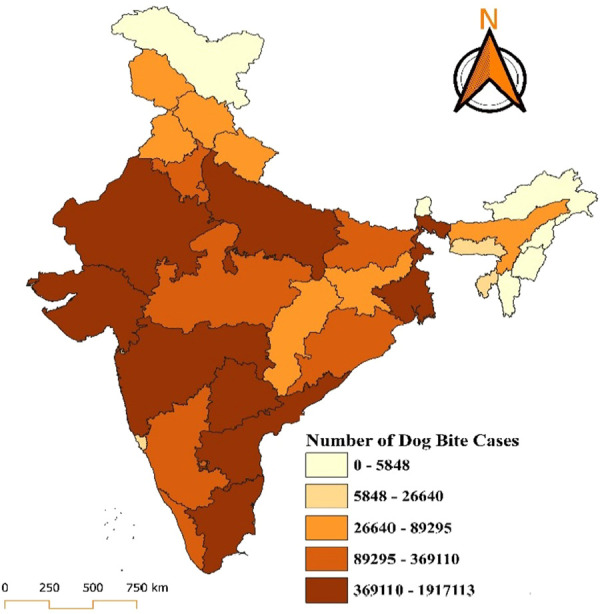
Distribution of dog bite cases in India for the year 2018.

**Figure 2.**
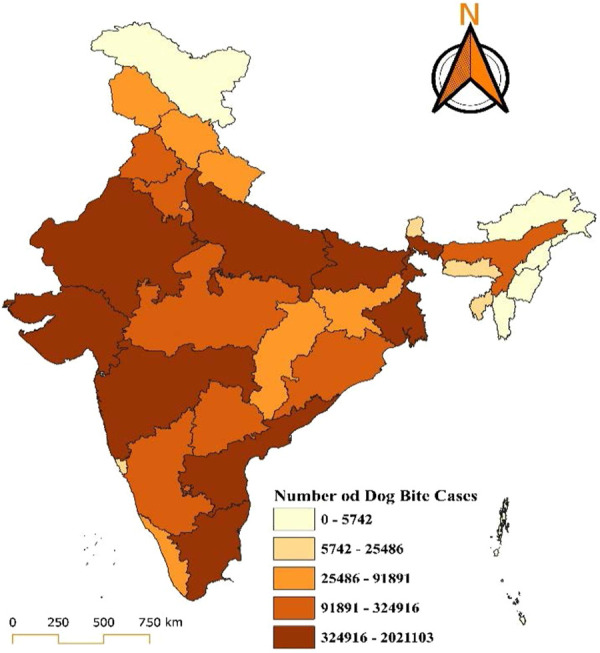
Distribution of dog bite cases in India for the year 2019.

The map provides a detailed visualization of the distribution of dog bite cases in India for the year 2018, showing significant regional disparities across states and union territories. The data are categorized into five ranges, each represented by a distinct colour gradient, with darker shades indicating a higher number of cases. The states with the highest burden, including Uttar Pradesh, Maharashtra, and Bihar, fall in the darkest brown category, reporting cases ranging from 3,69,110 to 1,917,113. States such as Madhya Pradesh, Rajasthan, Gujarat, Tamil Nadu, and West Bengal fall into the moderately high burden category, with reported cases ranging between 89,295 and 369,110. In the moderate-burden category, with cases ranging between 26,640 and 89,295, states such as Odisha, Haryana, Karnataka, Telangana, and parts of northeastern India show a relatively lower but still concerning number of cases. States like Kerala, Punjab, Himachal Pradesh, and Jammu and Kashmir, along with some northeastern states such as Arunachal Pradesh, fall into the low-burden category, with cases ranging between 5,848 and 26,640. The lowest burden, represented by the lightest yellow shade, includes union territories such as Lakshadweep, Andaman and Nicobar Islands, and states like Sikkim, with reported cases ranging from 0 to 5,848. Lakshadweep reported zero dog bite cases in 2018. In 2019, states such as Uttar Pradesh, Maharashtra, and Bihar reported the highest number of cases, ranging between 324,916 and 2,021,103, depicted in dark brown. These states remain critical hotspots for dog bite incidents, likely due to high population density, extensive stray dog populations, and limited access to vaccination and control programs. Moderately high-burden states, including Madhya Pradesh, Rajasthan, Gujarat, Tamil Nadu, and West Bengal, report cases ranging between 91,891 and 324,916. States such as Odisha, Telangana, Haryana, Karnataka, and Chhattisgarh fall into the moderate-burden range, with cases between 25,486 and 91,891. Regions like Kerala, Punjab, Himachal Pradesh, Jammu and Kashmir, and some northeastern states report relatively fewer cases, ranging from 5,742 to 25,486. The lowest burden of 0 to 5,742 cases, includes regions such as Lakshadweep, Andaman and Nicobar Islands, and Sikkim, with Lakshadweep notably reporting zero cases (Figs. 1 and 2).

**Figure 3.**
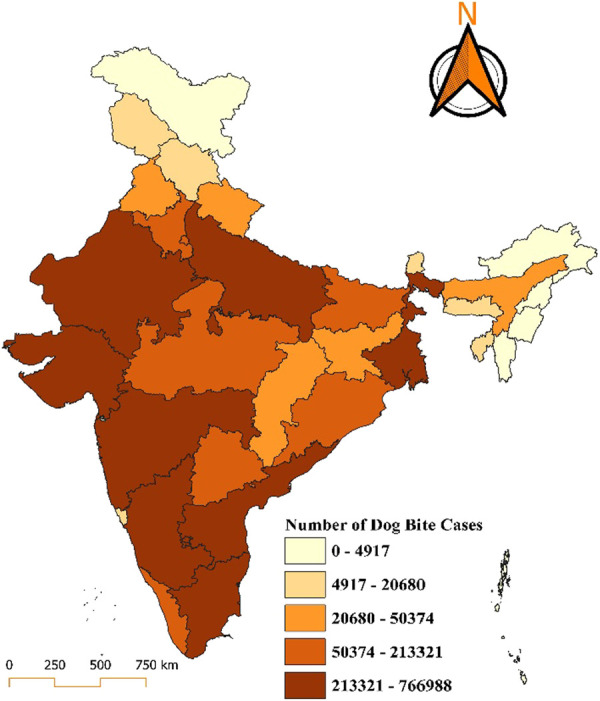
Distribution of dog bite cases in India for the year 2020.

In 2020, states with the highest burden, reporting between 213,321 and 766,988 cases, are prominently marked in dark brown and include Uttar Pradesh, Maharashtra, and Bihar. Moderately high-burden states, represented in lighter shades of brown, include Madhya Pradesh, Gujarat, Rajasthan, West Bengal, and Tamil Nadu, with reported cases ranging between 50,374 and 213,321. The middle category, with 20,680 to 50,374 cases, includes states like Haryana, Chhattisgarh, Odisha, Telangana, and parts of the northeast. States such as Kerala, Punjab, Himachal Pradesh, Jammu and Kashmir, and others fall into the lower-burden category, with 4,917 to 20,680 cases. The lowest category, reporting 0 to 4,917 cases, includes regions such as Lakshadweep, Andaman and Nicobar Islands, and Sikkim, with Lakshadweep notably maintaining a zero-case status (Fig. 3).

**Figure 4.**
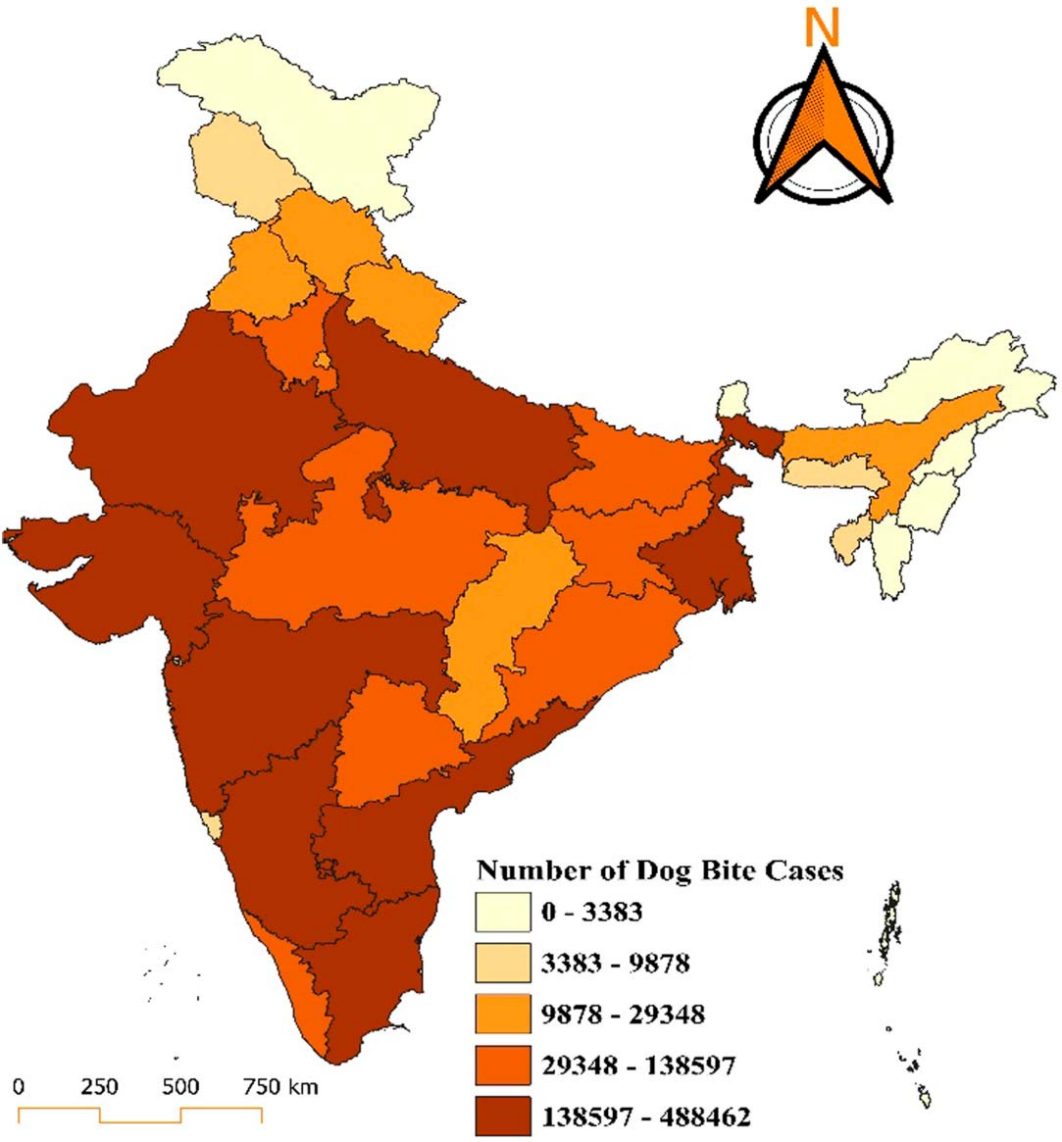
Distribution of dog bite cases in India for the year 2021.

In 2021 Uttar Pradesh and Madhya Pradesh, shaded in the darkest brown, represent the highest burden, with cases ranging from 138,597 to 488,462. States like Rajasthan, Bihar, Maharashtra, and Tamil Nadu, which fall into the medium-to-high burden category with cases between 29,348 and 138,597, face significant challenges as well. The moderate burden category, comprising states like Jharkhand, Chhattisgarh, Karnataka, Odisha, West Bengal, Telangana, and Andhra Pradesh, shows a case range of 9,878 to 29,348. States like Punjab, Haryana, Himachal Pradesh, Gujarat, and Assam, report relatively low cases (3,383–9,878 cases). The regions with the least burden (0–3,383 cases), including Jammu and Kashmir, Uttarakhand, Arunachal Pradesh, Sikkim, and the northeastern states along with Goa, Kerala, Lakshadweep, and the Andaman and Nicobar Islands, with Lakshadweep notably maintaining a zero-case status (Fig. 4).

**Figure 5.**
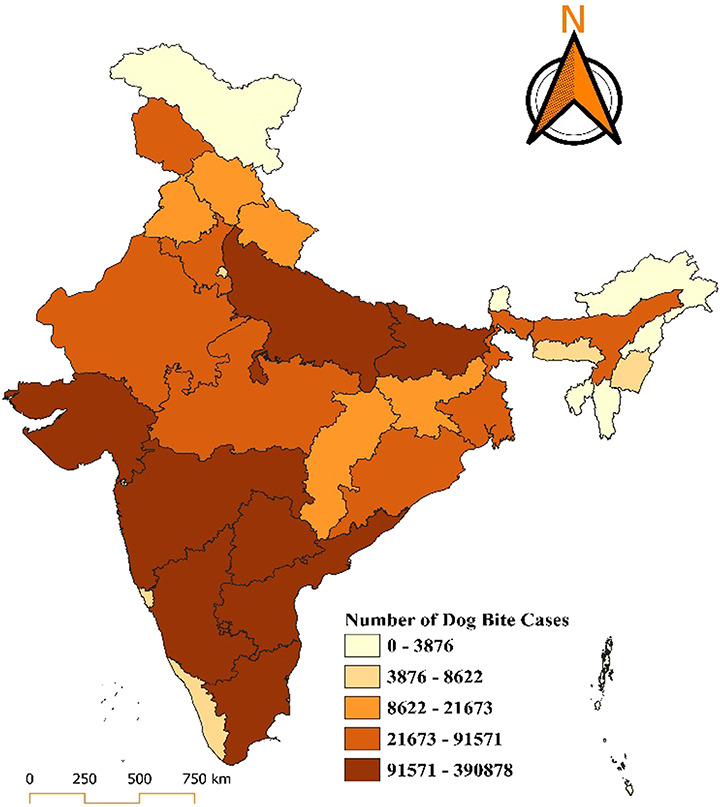
Distribution of dog bite cases in India for the year 2022.

In 2022, Uttar Pradesh and Madhya Pradesh, shaded in the darkest brown, continue to bear the highest burden, with cases ranging from 91,571 to 390,878. States such as Rajasthan, Bihar, Maharashtra, and Tamil Nadu, fall into the medium-to-high burden category 21,673 to 91,571 cases. Moderate burden regions like Jharkhand, Chhattisgarh, Odisha, Karnataka, Telangana, Andhra Pradesh, and West Bengal 8,622 to 21,673 cases. States such as Punjab, Haryana, Himachal Pradesh, Assam, and Gujarat, with a lower burden of 3,876 to 8,622 cases. Regions reporting the least cases (0–3,876), including Jammu and Kashmir, Uttarakhand, Arunachal Pradesh, and other northeastern states, as well as Goa, Kerala, and the Andaman and Nicobar Islands, with Lakshadweep notably maintaining a zero-case status (Fig. 5).

**Figure 6.**
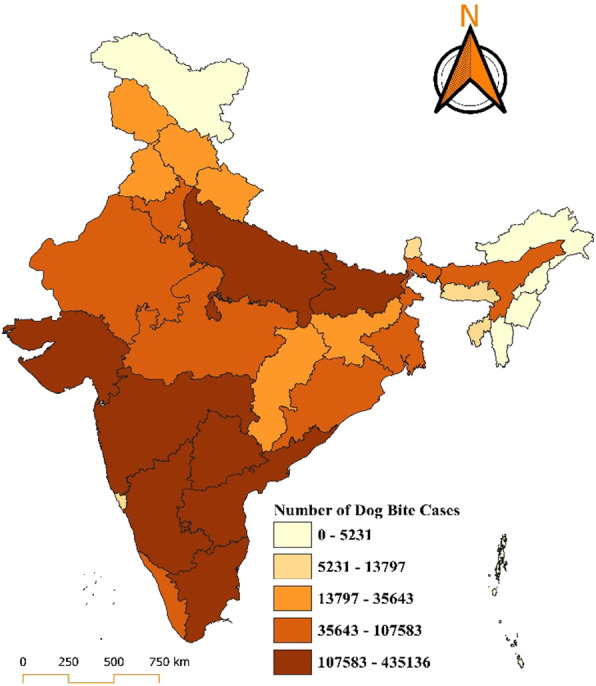
Distribution of dog bite cases in India for the year 2023.

In 2023, Uttar Pradesh and Madhya Pradesh continue to report the highest burden, with cases ranging from 107,583 to 435,136, indicated by the darkest brown on the map. States like Maharashtra, Rajasthan, Bihar, Tamil Nadu, and West Bengal, fall into the medium-high burden category of 35,643 to 107,583 cases. Regions such as Odisha, Jharkhand, Chhattisgarh, Telangana, Andhra Pradesh, Karnataka, and parts of Punjab, fall into the moderate range of 13,797 to 35,643 cases. States with a relatively lower burden of 5,231 to 13,797 cases, including Haryana, Himachal Pradesh, Assam, and Gujarat. The lowest range of 0 to 5,231 cases includes regions like Jammu and Kashmir, Arunachal Pradesh, other northeastern states, Goa, and the Andaman and Nicobar Islands, Lakshadweep notably maintaining a zero-case status (Fig. 6).

**Figure 7.**
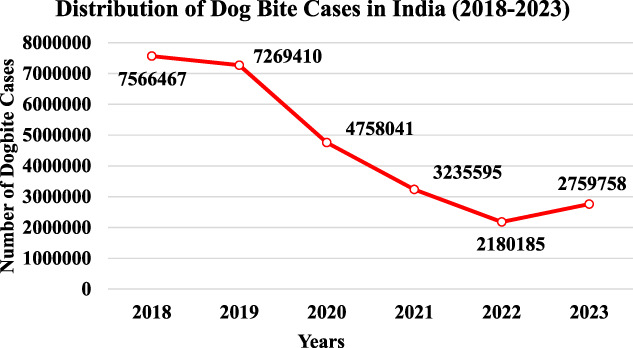


The data on dog bite cases in India from 2018 to 2023 shows a significant variation, indicating changing trends in the management and reporting of dog bites over the years (Fig. 7). In 2018, the number of reported cases was exceptionally high at 7,566,467, followed by a slight decline to 7,269,410 in 2019. This decline could reflect initial improvements in public health measures, stray dog population control, or underreporting in some areas. However, a sharp drop was observed in 2020, with cases plummeting to 4,758,041. This drastic reduction coincides with the COVID-19 pandemic, during which lockdowns and restricted movements may have contributed to reduced interactions between humans and stray dogs or disruptions in reporting systems.

The downward trend continued in 2021, with cases decreasing further to 3,235,595. This decline may reflect the residual effects of the pandemic or improved interventions such as sterilization and vaccination programs. By 2022, the number of cases reached its lowest point at 2,180,185, suggesting potential progress in controlling the issue through increased public awareness, better health care access, and preventive measures. However, a rise is observed in 2023, with cases increasing to 2,759,758. Overall, the data reveal a steady decline in dog bite cases from 2018 to 2022, followed by a slight rebound in 2023. While the decline over the years reflects some success in addressing the issue, the rise in 2023 highlights the need for sustained efforts, including intensified vaccination campaigns, sterilization drives, and community education programs, to ensure long-term control and prevention of dog bite cases in India.

## Discussion

The analysis of dog bite cases in India from 2018 to 2023 reveals a fluctuating trend, with an initial peak in 2018 (7.57 million cases) followed by a steady decline until 2022 (2.18 million cases) and a subsequent resurgence in 2023 (2.76 million cases). These variations suggest improvements in dog bite management but also highlight gaps in sustaining long-term interventions. Our findings align with a community-based study by Sudarshan et al (2006), which reported an annual dog bite incidence of 25.7 per 1,000 population, with higher rates among males and peaks during summer months. The study also found that 40% of bite victims did not seek medical care, indicating a significant issue of underreporting, which may partly explain the declining numbers in our dataset. Another study conducted in Delhi's urban and rural slums (Sharma et al, 2016) found a similar incidence rate of 25.2 per 1,000, with urban areas showing higher cases than rural regions, a trend that corresponds with our findings in states with high urbanization, such as Maharashtra and Tamil Nadu.^12,13^

The sharp decline in dog bite cases in 2020 and 2021 (4.76 million and 3.24 million, respectively) coincided with the COVID-19 pandemic, which limited human-dog interactions due to lockdowns. Saleem et al (2021) similarly reported reductions in reported bite cases during certain periods, although they cautioned that such declines might not reflect a true decrease in incidence but could instead be attributed to underreporting caused by disruptions in healthcare access. The resurgence in 2023 suggests either a genuine increase in dog bite incidents or improved reporting mechanisms postpandemic. Furthermore, our data indicate that Uttar Pradesh, Bihar, and Maharashtra remain persistent hotspots for dog bites, which aligns with epidemiological studies linking high stray dog populations, poor waste management, and inadequate vaccination programs to increased bite cases (Sharma et al, 2019). Seasonal variations also play a role, as previous studies (Sudarshan et al, 2007) have shown that dog bites peak during warmer months due to increased dog aggression and mating behaviors.^14^

India remains a global hotspot for rabies, with the WHO estimating that the country accounts for 36% of worldwide rabies deaths, with approximately 18,000–20,000 fatalities annually. A systematic review estimated around 17.4 million dog bites per year in India, suggesting significant underreporting in official records.^15^ Our findings indicate a positive trend in reducing dog bite cases between 2018 and 2022, possibly due to increased vaccination and sterilization efforts. However, the rise in cases in 2023 highlights the need for sustained interventions. Strengthening anti-rabies vaccination coverage, enhancing sterilization programs to control stray populations, improving public awareness on postbite wound care, and establishing robust surveillance systems for better reporting are critical to sustain progress. While India has made notable progress in dog bite management, the resurgence in 2023 emphasizes the importance of continuous and well-coordinated public health initiatives to prevent future outbreaks and reduce the burden of dog bites and rabies-related deaths.

## Limitations

This study offers valuable insights into dog bite trends in India (2018–2023) but has key limitations. It relies on reported cases, likely underestimating the burden due to underreporting, especially in rural areas. Inconsistent data collection across states and the impact of the COVID-19 pandemic further affect accuracy. The study also does not assess factors like seasonal trends, stray dog populations, or effectiveness of control programs nor does it consider socioeconomic and environmental influences limiting its ability to identify causal factors.

## Conclusion

Dog bite cases in India declined from 2018 to 2022 due to vaccination, sterilization, and awareness programs, but increased in 2023, highlighting the need for sustained efforts. High-burden states like Uttar Pradesh, Bihar, and Maharashtra require targeted interventions. Effective surveillance, timely treatment, and community engagement are crucial for reducing cases and preventing rabies. Strengthening vaccination, waste management, and access to PEP are key to long-term control.
